# Compartmentalized organization of ecological niche occupation in insular invertebrate communities

**DOI:** 10.1002/ece3.7067

**Published:** 2020-11-24

**Authors:** Sebastian Steibl, Christian Laforsch

**Affiliations:** ^1^ Department of Animal Ecology and BayCEER University of Bayreuth Bayreuth Germany

**Keywords:** ecological community, habitat, insular ecosystem, modularity, Niche clustering, niche segregation, species assemblage

## Abstract

Understanding the mechanisms of species distribution within ecosystems is a fundamental question of ecological research. The current worldwide changes and loss of habitats associated with a decline in species richness render this topic a key element for developing mitigation strategies. Ecological niche theory is a widely accepted concept to describe species distribution along environmental gradients where each taxon occupies its own distinct set of environmental parameters, that is, its niche. Niche occupation has been described in empirical studies for different closely related taxa, like ant, ungulate, or skink species, just to name a few. However, how species assemblages of whole ecosystems across multiple taxa are structured and organized has not been investigated thoroughly, although considering all taxa of a community would be essential when analyzing realized niches. Here, we investigated the organization of niche occupation and species distribution for the whole ground‐associated invertebrate community of small tropical insular ecosystems. By correlating environmental conditions with species occurrences using partial canonical correspondence analysis (pCCA), we demonstrated that the ground‐associated invertebrate community does not spread evenly across the overall niche space, but instead is compartmentalized in four distinct clusters: crustacean and gastropod taxa occurred in one cluster, attributable to the beach habitat, whereas hexapods and spider taxa occurred in three distinct inland clusters, attributable to distinct inland habitats, that is, grassland, open forest, and dense forest. Within the clusters, co‐occurrence pattern analysis suggested only a few negative interactions between the different taxa. By studying ground‐associated insular invertebrate communities, we have shown that species distribution and niche occupation can be, similar to food webs, organized in a compartmentalized way. The compartmentalization of the niche space might thereby be a mechanism to increase ecosystem resilience, as disturbances cascade more slowly throughout the ecosystem.

## Graphical abstract

While niche occupation has been thoroughly studied in co‐occurring closely related taxa (e.g., scorpions, ungulates, dolphins, etc.), we present the first community‐wide analysis of niche occupation. Studying the niche occupation patterns of the ground‐associated invertebrate fauna on small tropical islands, we show that niche occupation is organized in a compartmentalized way. Instead of spreading evenly/ homogeneously over the overall niche space, species cluster into distinct niche compartments. This compartmentalization into niche clusters is likely caused by niche conservatism, where closely related taxa occupy more similar niches.

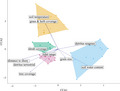

## 1 INTRODUCTION

The central goal of ecology is to understand species interactions with the biotic and abiotic environment. In the light of growing human land demands, it becomes increasingly relevant to predict species’ interactions and responses to the accelerating environmental changes (Holt, 2009). Therefore, understanding species distribution and the role of environmental variability is considered to be among the most urgent and fundamental goals for ecological research (Sutherland et al., 2013). Characterizing those factors that shape animals’ distribution in a given ecosystem will ultimately protect the habitat features necessary for a species’ persistence (Broennimann et al., 2012).

One of the most widely accepted concepts to describe species distribution in the environment is niche theory, originally postulated by Hutchinson in 1957 and continuously updated and extended following recent empirical research and modeling. Hutchinson (1957) stated that species distribute in the environment according to their ecological niche. A species’ ecological niche is defined as the n‐dimensional hypervolume that comprises all biological, chemical, and physical parameters of a heterogeneous environment in which a species can exist indefinitely. Differentiations are made between the fundamental niche of a species, which is the set of environmental conditions in which a species theoretically can live and reproduce in, and the much narrower realized niche, which contains the set of conditions that a species occupies, including its biological interactions with other species. The axes in this abstract model niche space correspond to the environmental factors that influence the organisms’ performance and incorporate, in general, habitat, diet, and time (Holt, 2009; Kiszka et al., 2011).

The gradient of each environmental factor in a given ecosystem is the key determinant of niche occupation and ecosystem organization. As species distribute along the environmental gradients of an ecosystem according to their specific niche optimum, heterogeneous ecosystems have more available niche space (Schwilk & Ackerly, 2005). They can, therefore, carry an overall higher number of species (Chesson & Warner, 1981; Harner & Harper, 1976; Kadmon & Allouche, 2007). High diversity and abundance of species mean that the distance between the niche optima of different species along a fixed environmental gradient decreases, which results in an even spacing of species across the heterogeneous environment (D’Andrea & Ostling, 2016; Schwilk & Ackerly, 2005).

Simultaneously, for low‐diversity systems, an increase in niche space due to heterogeneity results in potentially empty niche space allowing for more variation in the spacing of species (D’Andrea & Ostling, 2016). Especially when suitable conditions occur within a larger set of less favorable conditions, this can result in the formation of species aggregations or clusters (Fox, 1981). These clusters in niche space are formed by species that require similar environmental conditions, that is, similar niche optima, while species with different niche optima are organized in different clusters with little to no overlap (Goodman, 2007).

However, studying niche occupation and cluster formation in natural ecosystems is challenging (Darmon et al., 2012). Empirical studies have mainly focused on closely related taxa, like ants (Goldstein, 1975), dolphins (Kiszka et al., 2011), spiders (Entling et al., 2007), skinks (Goodman, 2007), ungulates (Darmon et al., 2012), scorpions (Goodman & Esposito, 2020), or peracarid crustaceans (Lastra et al., 2009), just to name a few. This gives relevant insight into the mechanisms of coexistence and differentiation between closely related taxa but generates only a limited understanding of the organization and architecture of whole faunal communities within an ecosystem. As the realized niches of species within ecosystems depend on the interactions with all other co‐occurring taxa (Hutchinson, 1957), including all taxa of a given ecosystem would be essential when investigating the architecture of niches and their occupation in ecosystems.

Empirically, this can best be achieved in simple communities as they occur on islands (MacArthur & Wilson, 1967). The key advantage of insular ecosystems is that ecological processes can be observed more comprehensively than on continental, mainland ecosystems (Goldstein, 1975). Additionally, the overall smaller size, distinct boundaries formed by the adjacent ocean, and reduced species richness allow us to observe and interpret the patterns of niche occupation better and include multiple taxa (Losos & Ricklefs, 2009; MacArthur et al., 1972).

Here, we used small tropical insular ecosystems to investigate the organization of niche occupation and species distribution of the whole ground‐associated faunal community. Because in the investigated system, the Maldivian archipelago, vertebrate taxa are virtually absent (except for only locally common sea birds, two species of flying foxes, two species of amphibians, and five species of reptiles), the study focused on the ground‐associated invertebrate community. We hypothesized that different small tropical islands in the same region provide the overall same ecological niche space for the present ground‐associated invertebrate taxa and that these taxa cluster in distinct niche patches due to the overall low diversity. We examined the relevant environmental gradients for the distribution patterns and tested whether co‐occurrence patterns appear within the identified patterns indicating mechanisms of niche partitioning or competitive exclusion.

## 2 METHODS

### 2.1 Field sampling

**Figure 1:**
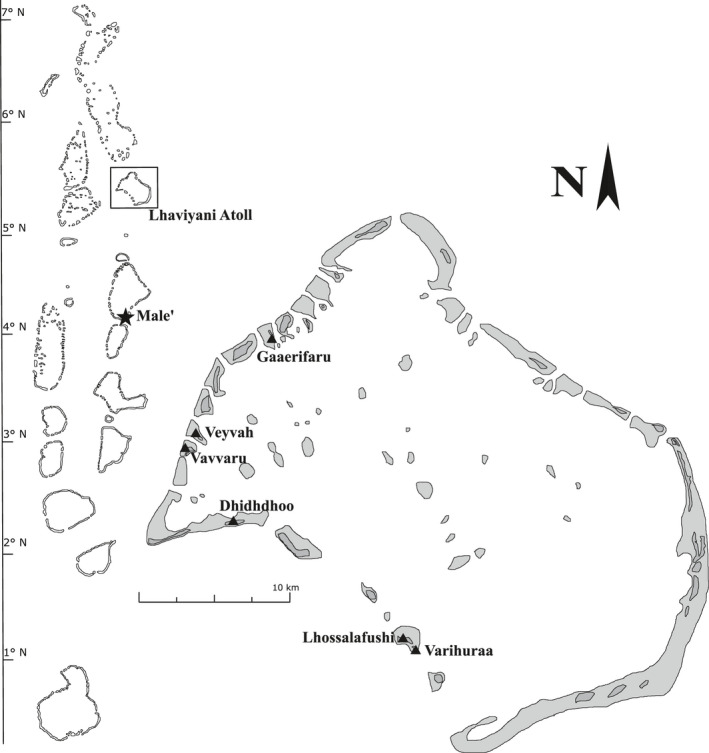
Position of the Lhaviyani (Faadhippolhu) atoll within the Republic of the Maldives (left) and location of the sampled islands, Dhidhdhoo, Gaaerifaru, Lhossalafushi, Varihuraa, Vavvaru, and Veyvah (right). Dark gray indicates land masses, and light gray around the islands indicates the spatial extensions of the lagoons and reefs surrounding each coral island. Note that Lhossalafushi and Varihuraa are two separate islands that do not share any land bridge but have the same outer coral reef

All sampling was carried out between 21 February 2019 and 4 April 2019, sampling one island per day on six uninhabited tropical islands in the Lhaviyani (Faadhippolhu) Atoll, the Republic of Maldives (Figure 1). The island sizes were estimated using GPS by walking along the shoreline of each island (Garmin eTrex Vista Cx; Garmin International Inc., Olathe, USA). The six islands’ circumferences were as follows: Dhidhdhoo: 2,400 m, Gaaerifaru: 862 m, Lhossalafushi: 2,610 m, Varihuraa: 645 m, Vavvaru: 855 m, and Veyvah: 706 m.

For each sampling day, the weather conditions and the tidal range, that is, the height difference between current water level and neap tide [m], were noted. On each island, 20 1 x 1 m‐plots were distributed randomly over the whole island area by placing a grid over each island's map and randomly selecting 20 sampling grids (*N* = 20). On each plot, a picture from a height of 1.80 m downwards and a picture facing skywards was taken to estimate vegetation coverage (D5000, Nikon Corp.). The exact location was marked using GPS, and the sampling time was noted. All present and visible day‐active ground‐associated invertebrate taxa in the plot were identified to the lowest possible taxonomic level (species or genus) using available identification literature and counted. Interstitial and soil‐associated invertebrate taxa were not included in the sampling. Ghost crab abundance (*Ocyode cordimana*) was measured by counting the number of burrows within each plot (Rodrigues et al., 2016). Afterward, all debris and detritus present in the plot were collected, assigned to either of the two categories “seagrass” (i.e., leaves of *Caulerpa spp., Posidonia spp., Syringodium spp., Thalassia spp*.), or “terrestrial” (i.e., leaves of autochthonous terrestrial plants and small deadwood), and weighed on‐site using a fine scale (NTP2K 2,000 ± 0.1 g, Nohlex GmbH, Buchholz, Germany). The soil temperature was measured on the four edges of the plot at a depth of 1.4 cm using a digital precision thermometer (P300W 0–100 ± 0.5°C, Dostmann electronic GmbH, Wertheim‐Reicholzheim, Germany) and averaged for each plot. The distance of the center of each plot to the nearest shoreline was measured. A soil sample from each plot was taken by scraping off the top 3 cm layer in a 10 × 10 cm area at each of the plot's four corners using a metal shovel. The soil samples were weighed and dried until no further weight reduction. When fully dried, the soil samples were weighed again and the delta value, that is, the soil water percentage, of each plot calculated. The fully dried soil samples were fractionated through a sieve combination, the weight in each fraction (6.3, 2, 0.63, 0.1 mm) was measured, and the mean grain size calculated from the proportional weights in each fraction was recorded. The percentage of grass/herb, shrub, and tree coverage was measured for each plot using ImageJ 1.49b (Rasband, W.S., ImageJ, U. S. National Institutes of Health, Bethesda, Maryland, USA, http://imagej.nih.gov/ij/, 1997–2015).

### 2.2 Statistical analysis

All statistical analyses were carried out using R 3.5.3 (R Core Team, 2018) extended with the “vegan” package (Anderson, 2001).

#### 2.2.1 Similarity of overall niche space (NMDS)

The islands’ overall ecological niche space was compared between the investigated islands (*N* = 6) using nonmetric multidimensional scaling (NMDS). NMDS is a robust ordination technique used to compare differences in parameter compositions among multiple sites (Oksanen, 2015). Prior to NMDS analysis, the investigated physical parameters (soil temperature [°C], soil grain size [mm], seagrass detritus amount [g], terrestrial detritus amount [g], soil water content [%], grass & herb coverage [%], shrub coverage [%], tree coverage [%]) were rescaled between 0 and 1. The output of the *k* = 2 dimension NMDS representation showed high regression between ordination distances and community dissimilarities (*R* ^2^ = 0.964). To statistically test for differences in the physical parameter set between the investigated islands, nonparametric multivariate analysis of variances (PERMANOVA) with post hoc testing using Bray–Curtis dissimilarity indices and 4,999 permutations was performed.

#### 2.2.2 Species distribution in the niche space (pCCA)

NMDS and PERMANOVA testing indicated that the investigated islands generally provide the overall same ecological niche space (*p* > .05 for all but one pairwise comparison; see also results section). The obtained physical parameters and species abundance matrices (plot × species) were pooled over the six islands for subsequent partial canonical correspondence analysis (pCCA). This was further necessary, as calculating a separate pCCA for each island would not allow for any comparability between the islands as the loadings of the pCCA axes differ between each analysis (and hence island). The pCCA treats the physical parameter data matrix as the predictor and the plot x species abundance matrix as the response in multivariate multiple regression, where the gradient axes are constrained as linear combinations of the environmental variables (Ter Braak, 1987). An advantage of pCCA is that the effect of specific, redundant environmental parameters can be excluded, thereby allowing us to merely investigate the effects of those physical parameters that are of primary interest while controlling for other spatial between‐island differences, for example, distance between systems (Reiskind et al., 2017). Abundance data were log + 1 transformed to dampen effects of dominant or very rare taxa. A pCCA was run with the plot x species abundance matrix as the response, the rescaled physical parameters (soil temperature, soil grain size, seagrass detritus amount, terrestrial detritus amount, soil water content, grass & herb coverage, shrub coverage, tree coverage, distance to the nearest shore, tidal range, rescaled to range between zero and one) as predictor variables and the spatial data (longitudinal and latitudinal position of each plot; obtained from GPS) as the conditioning variables. Variance inflation factors (VIF), which indicate collinearity between predictor variables, scored VIF < 4 for all predictors, thus showing no problematic redundancy in the variable set. Permutation tests for constrained correspondence analysis (999 permutations) were performed to test whether the pCCA model, the physical parameters (predictor variables), and the pCCA axes significantly explain the variance in the plot x species abundance matrix (response variables).

#### 2.2.3 Cluster formation within the niche space (Cluster analysis)

Permutation tests suggested that CCA1 and CCA2 significantly explain variance in the plot × species abundance matrix (*p* < .05; see also results section). Therefore, scores from the first two CCAs were used for subsequent analysis. Cluster analysis was performed using scores of CCA1 and CCA2 of each taxon to test for any underlying compartmentalized structure in niche occupation. NbClust method, which uses 30 different indices for determining the most likely number of clusters *K*, was conducted (Charrad et al., 2014). To test, whether the clusters differ statistically in their pCCA scores, we calculated the mean pCCA1 and mean pCCA2 score of each cluster by averaging all taxa scores assigned to the particular cluster. The mean pCCA scores were statistically compared between the identified clusters using ANOVA with TukeyHSD post hoc testing.

#### 2.2.4 Co‐Occurrence pattern analysis

The abundance data matrix was subsequently subset into the identified clusters. For each of the clusters, an additional pCCA following the same procedure as described above was performed. To investigate patterns of co‐occurrence within the identified clusters, we used the “cooccur” package (Griffith et al., 2016). Before analysis, the abundance data matrices of the identified clusters were transformed into presence–absence matrices. For all taxa pairs in each of the identified clusters, the “cooccur()” function produces probabilities of co‐occurrence, which are greater or less than those observed in the sampling. To enumerate all possible species combinations within the investigated clusters, the implemented threshold that allows us to investigate only the most important associations (i.e., remove those species pairs expected to share less than one site) was used. The output of the analysis is distribution‐free, and the probabilities can be considered as p‐values, which indicate whether two taxa are significantly negatively or positively associated with their occurrence (for details on calculations refer to Griffith et al. (2016)).

## 3 RESULTS

### 3.1 Comparison of overall niche space of the investigated insular ecosystems

**Figure 2:**
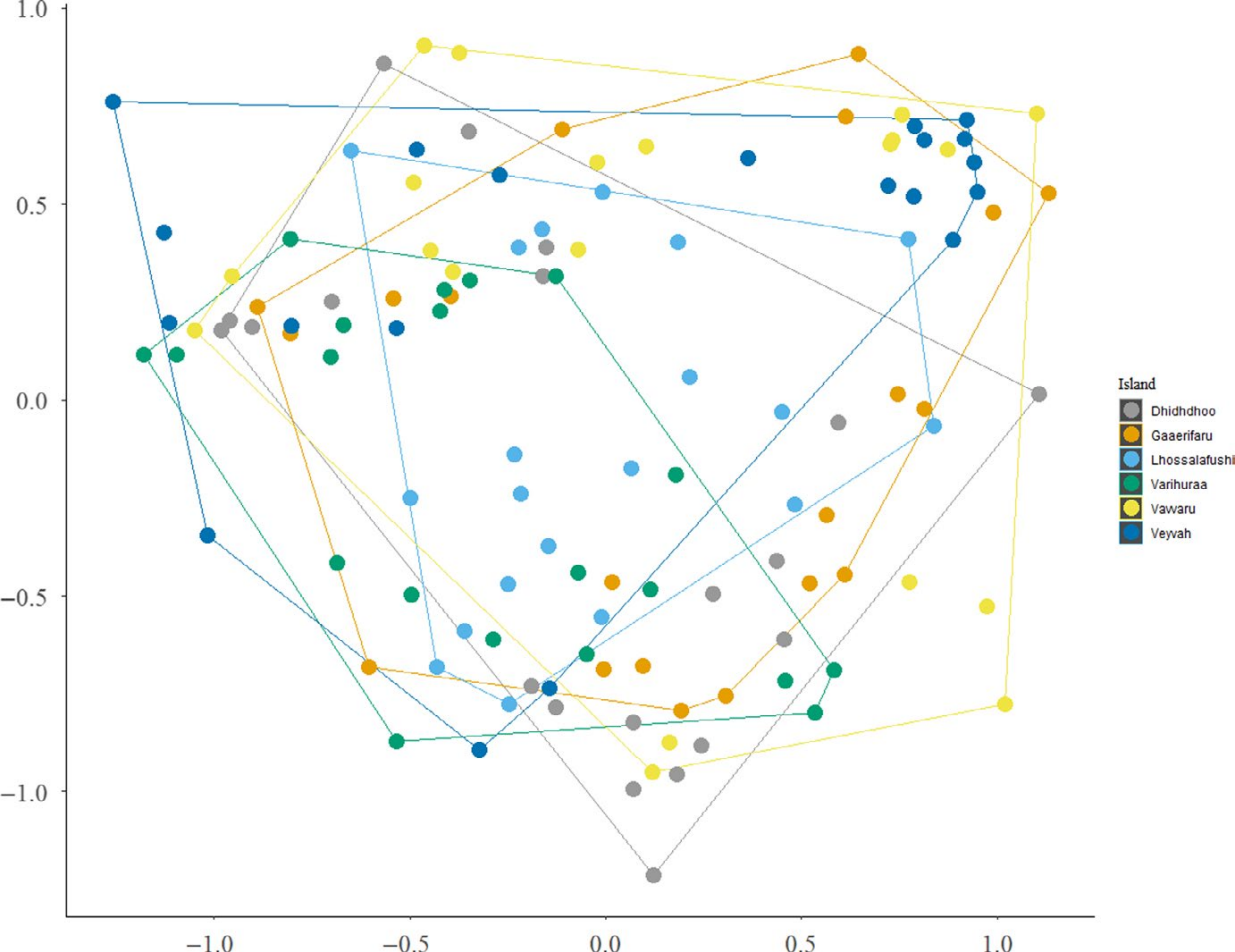
Ecological niche space provided by the investigated uninhabited coral islands (*N* = 6). Calculation of the NMDS representation of the niche space is based on normalized values for the following parameters: soil temperature, soil grain size, seagrass detritus amount, terrestrial detritus amount, soil water content, grass & herb coverage, shrub coverage, tree coverage. Each data point represents one plot (*N* = 20 per island), and different colors indicate different islands. The spatial proximity of any two data points in the NMDS representation indicates the similarity of the two plots

Overall, the six investigated tropical islands provided, in general, the same physical parameters (PERMANOVA: all pairwise comparisons *p* > .05, except Veyvah – Lhossalafushi: *F *= 8.408, *p *= .015) (Figure 2).

### 3.2 Species distribution along the environmental parameters and cluster formation

**Figure 3:**
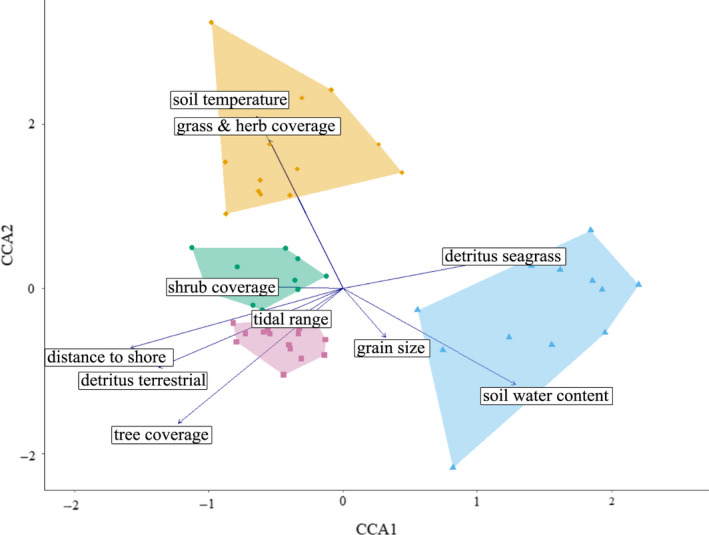
CCA representation of the species distribution within the ecological niche space of the investigated uninhabited islands (CCA model: *F* = 1.990, *p* = .001; for details on model performance refer to Table 1). Each data point represents a single taxon within the ecological niche space, and different colors and hulls indicate the cluster assignments (NbClust method. blue: beach cluster, yellow: grassland cluster, green: open forest cluster, purple: dense forest cluster; see also Table S1 for detailed taxa identities of each cluster). The spatial proximity of any data point to a physical parameter vector indicates that this parameter influences the distribution/occurrence of the particular taxon. The vectors of physical parameters that point in the same direction indicate a positive correlation between them, and vectors that point in the opposite direction indicate a negative correlation between them

The pCCA output revealed that the investigated physical parameters explained a significant amount of variation in the ground‐associated invertebrate community (permutational test for constrained correspondence analysis: *F* = 1.990, *p* < .001) (Figure 3; for an overview of all identified taxa refer to appendix Table S1). After controlling for spatial covariables, the investigated physical parameters explained 16.06% of the overall variation in the species distribution. Permutational tests suggested that the first two CCAs significantly explained species distribution (CCA1: *F *= 6.209, *p* < .001; CCA2: *F* = 3.298, *p* = .035; Table 1). CCA1 thereby accounted for 31.20% of the total explained variance (eigenvalue: 0.695) and CCA2 for 16.58% (eigenvalue: 0.369). Most obtained physical parameters thereby significantly explained species distribution (Table 1).

**Table 1:** Summary of the pCCA output and statistical testing of the physical parameters (predictor variables) and CCA axes based on permutational tests (999 permutations)

	Explained variance	*F*‐Value	*p‐*Value
Overall CCA model	‐	1.990	.001***
Tidal range	‐	1.396	.047*
Shore distance	‐	3.200	<.001***
Soil temperature	‐	3.028	<.001***
Detritus seagrass	‐	1.110	.312
Detritus terrestrial	‐	2.553	.003**
Soil water content	‐	2.197	.002**
Grain size	‐	1.719	.101
Grass herb coverage	‐	1.814	.030 *
Shrub coverage	‐	0.955	.487
Tree coverage	‐	1.923	.005**
CCA1	31.20%	6.209	<.001***
CCA2	16.58%	3.298	.035*
CCA3	12.02%	2.392	.202
CCA4	10.04%	1.998	.353
CCA5	7.51%	1.496	.764
CCA6	6.43%	1.281	.880
CCA7	6.19%	1.233	.821
CCA8	4.64%	0.924	.968
CCA9	3.24%	0.645	.993
CCA10	2.11%	0.420	.982

Note

Asterisks indicate significance levels *0.05; **0.01; ***0.001.

Cluster analysis revealed that the present taxa do not spread homogenously over the provided niche space, but rather that *K* = 4 clusters occur within the niche space of the investigated islands. The four identified clusters differed significantly in their mean CCA scores (Table 2). Cluster 1, which comprises most crustacean and all molluscan taxa present on the investigated islands, showed a significantly higher mean score in CCA1 than clusters 2, 3, and 4, which comprised all hexapod and insect taxa (Tukey HSD: *p* < .001 for all pairwise comparisons). CCA1 is represented by high soil water and seagrass detritus amounts, proximity to the shoreline, as well as low tree coverage and terrestrial detritus amounts. Therefore, cluster 1 could be attributed to the beach habitat and considered the “beach cluster,” as those taxa occurred in proximity to the shore and where seagrass was abundant. Cluster 4 showed a significantly higher mean score in CCA2 than clusters 1–3 (Tukey HSD: *p* < .001 for all pairwise comparisons). CCA2 is represented by high soil temperature and grass/herb coverage scores, as well as low soil water content and tree coverage. Therefore, cluster 4 can be attributed to the grassland habitat and considered the “grassland cluster,” as those taxa occurred in dry areas with high grass coverage. Cluster 2 and 3 are both distinct by having low scores in CCA1 (Tukey HSD: *p* = .999), that is, occurring preferably in areas with high tree coverage and terrestrial detritus and being further inland. Cluster 2 had a significantly higher score in CCA2 than cluster 3 (Tukey HSD: *p* = .004), suggesting that taxa in cluster 2 occurred in forested areas with understory grass & herb vegetation, while taxa in cluster 3 occurred in the inland with denser tree coverage and no understory vegetation. Therefore, cluster 2 can be attributed to the open and dense forest habitats and considered the “open forest cluster” and cluster 3 the “dense forest cluster.”

**Table 2:** The relative contribution of the investigated physical parameters (predictor variables) to CCAs and mean (± *SD*) CCA1 and CCA2 score for the four identified clusters

	**CCA1**	**CCA2**
Tidal range	−0.112	−0.113
Shore distance	**−0.631**	−0.263
Soil temperature	−0.239	**0.820**
Seagrass detritus	**0.435**	0.131
Terrestrial detritus	**−0.551**	−0.352
Soil water content	**0.497**	**−0.455**
Soil grain size	0.122	−0.239
Grass/ herb coverage	−0.206	**0.726**
Shrub coverage	−0.248	−0.032
Tree coverage	**−0.500**	**−0.605**
Cluster 1 (beach cluster)	1.753 ± 0.613 (B)	−0.461 ± 1.212(AB)
Cluster 2 (open forest cluster)	−0.611 ± 0.376 (A)	0.408 ± 0.320 (B)
Cluster 3 (dense forest cluster)	−0.601 ± 0.282 (A)	−0.878 ± 0.352 (A)
Cluster 4 (grassland cluster)	−0.484 ± 0.507 (A)	2.862 ± 1.206 (C)

Note

Scores in bold identify the main predictors for the respective CCA axes. Different letters after the CCA scores of the four clusters indicate significant differences in the CCAs in the pairwise comparisons of the clusters (ANOVA, Tukey HSD).

### 3.3 Co‐occurrence analysis within the identified niche compartments

To investigate differences within the four identified clusters, pCCA was performed for each cluster. The physical parameters were no longer able to describe variations in the distribution within the cluster 2, that is, “open forest cluster” (*F* = 1.023, *p* = .429), cluster 3, that is, “dense forest cluster” (*F *= 0.936, *p* = .569), and cluster 4, that is, “grassland cluster” (*F* = 1.174, *p* = .162). For cluster 1, that is, “beach cluster” the physical parameters significantly explained variations in species abundance (*F* = 2.026, *p* = .006). Permutational tests, however, suggested that none of the CCA niche axes could explain species distribution (CCA1: *F* = 6.942, *p* = .076; CCA2: *F *= 5.573, *p* = .119; CCA3: *F* = 2.957, *p* = .523; CCA4: *F* = 2.332, *p* = .657).

**Figure 4:**
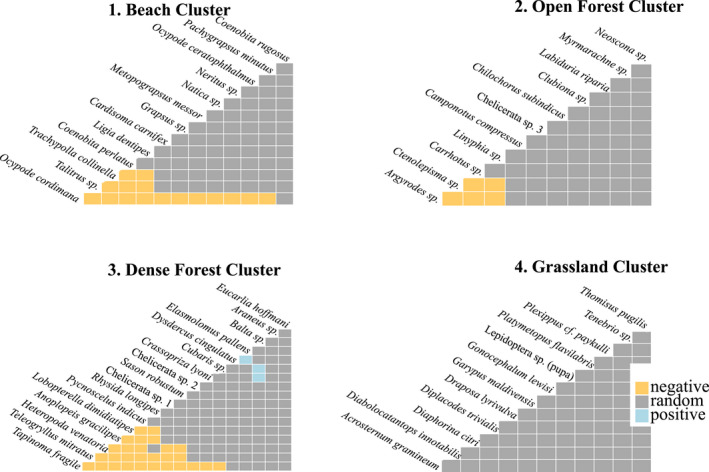
Co‐occurrence pattern analysis within the four identified clusters 1–4 (see also Table S1). Negative association, that is, occurrence of taxon 1 excludes taxon 2, between two taxa pairs indicated by yellow rectangles, positive co‐occurrence, that is, occurrence of taxon 1 favors occurrence of taxon 2, indicated by blue rectangles and random co‐occurrence, that is, no significant positive or negative association between two taxa, by gray rectangles

Analysis of the co‐occurrence patterns for each of the four identified clusters suggests that significantly negative associations between species pairs occur in cluster 1 (“beach cluster”), in cluster 2 (“open forest cluster”), and cluster 3 (“dense forest cluster”) (Table 3, Figure 4). A significant positive association was identified between species pairs in cluster 3 (“dense forest cluster”), that is, a cockroach (*Balta sp*.), woodlice (*Cubaris sp*.), and a carpenter spider (*Crassopriza lyoni*), as well as a beetle species (*Elasmolomus pallens*) and a bug (*Dysdercus cingulatus*) (Figure 4c). No significant positive or negative association between any taxon pair was found in cluster 4 (“grassland cluster”).

**Table 3:** Summary of the co‐occurrence analysis of taxa within the four identified clusters

	**Cluster 1 (Beach)**	**Cluster 2 (Open Forest)**	**Cluster 3 (Dense Forest)**	**Cluster 4 (Grassland)**
Positive associations	0	0	3	0
Negative associations	16	5	28	0
Random associations	75	61	122	78
Nonrandom associations	18.7%	7.6%	20.3%	0%

Note

Associations were determined as “random” when pairs did not differ significantly from the expected number of co‐occurrences and deviated <10% of the total number of sites.

## 4 DISCUSSION

Most previous empirical studies on niche occupation focused on closely related taxa and did not explicitly consider the entire community. Here, we studied the structure and occupation of the ecological niche space of the entire ground‐associated invertebrate community on small tropical atoll islands in the Indo‐Pacific. Analysis of the occupied ecological niche space suggested that the investigated taxa do not spread evenly over the provided niche space, but instead aggregated in four distinct clusters within the overall niche space, attributable to habitats within the low‐lying atoll insular ecosystem, that is, beach, grassland, open forest, and dense forest. The majority (~85%) of all associations between taxa pairs within each cluster showed no negative interactions, indicating that neither mechanisms of competitive exclusion nor contrasting habitat preferences dominate the interactions within the clusters. In contrast, only a few taxa appear to be positively or negatively associated with one another.

Abiotic (i.e., tidal range, shore distance, soil temperature, soil water content) and biotic (i.e., terrestrial detritus, grass & herb coverage, tree coverage) parameters significantly influenced the distribution of the ground‐associated invertebrate community on the investigated low‐lying atoll islands in the Indo‐Pacific. The same set of parameters influenced animals’ occurrences across multiple taxa, demonstrating that the factors and mechanisms that cause niche occupation and differentiation are consistent not only within closely related taxa as shown, for instance, in spiders (Entling et al., 2007), ungulates (Darmon et al., 2012), or dolphins (Kiszka et al., 2011) but also across species assemblages of whole insular invertebrate communities. Although a high proportion of variation in species occurrence remained unexplained in the conducted pCCA model in this study, the investigated environmental parameters were able to significantly explain species distribution over a wide range of unrelated taxa and indicate clustering mechanisms within the available niche space. Our data indicate that the major environmental parameters (e.g., temperature, humidity, vegetation cover, etc.) only contribute to a small proportion of the overall occurrence patterns of faunal communities, that is, roughly 16%. Trophic relationships, inter‐ and intraspecific competition, or pure stochasticity could, therefore, be equally or even more relevant determinants of insular invertebrate community assemblages than environmental parameters.

According to Hutchinsons (1957) original postulations on ecological niches, the present study confirmed that niche differences are essential in structuring the occurrence of closely related taxa and whole invertebrate communities. Faunal communities on remote and low‐lying tropical atolls islands are dependent on various physical and chemical parameters, type of vegetation cover, and distance to the shoreline. However, the analyzed taxa did not spread evenly across the overall ecological niche space, but instead aggregated in four distinct clusters, that is, beach, grassland, open forest, and dense forest. This clustering into distinct habitats might be a crucial mechanism that allows coexistence, even in small and remote insular ecosystems with overall limited space (Chesson, 2000; Goodman, 2007). The clustering could further be favored by the overall low diversity in these remote and low‐lying atoll insular ecosystems because low‐diversity systems offer relatively more available niche space to each species (D'Andrea & Ostling, 2016; Fox, 1981).

A conspicuous feature of the clustering within the niche space in our study is that most identified crustacean and all molluscan taxa occurred exclusively in the beach cluster, whereas most hexapods and all spider taxa only occurred in the three inland clusters, that is, grassland, open forest, and dense forest. Both observations might be caused by niche conservatism, where the realized ecological niches of related taxa remain similar due to only a slow evolution of niche occupation and differentiation (Peterson et al., 1999; Wiens et al., 2010). As beach‐associated crustaceans colonized the terrestrial environment from the adjacent ocean and are still closely associated to the shoreline in parts of their life cycle, for example, for reproduction, many of their adaptations, for example, returning to the shore for spawning, are conserved across different taxa (Bliss & Mantel, 1968; Greenaway, 2003; Harzsch et al., 2011; Taylor, 1988). For hexapod and spider taxa in the inland clusters, adaptations to terrestrial life in the inland might hinder distribution and differentiation into the beach habitat, as conditions require specific adaptations to withstand, for example, heat, high soil water content, or soil salinity on the beaches (Defeo & McLachlan, 2005). Therefore, niche conservatism might explain why closely related taxa clustered within the niche space and did not spread randomly over all four clusters.

Within each of the four identified clusters, the respective taxa overlapped in their occurrence and co‐occurrence pattern analysis within each cluster indicated that the majority of taxa (85% of all pairwise comparisons) co‐occur without any negative associations, like competitive exclusion or contrasting habitat preferences (Griffith et al., 2016). Co‐occurrence can be achieved when resources are abundant and not limiting (Darmon et al., 2012; Fox, 1981). In the investigated tropical insular ecosystem in the Indo‐Pacific, sufficient resource availability inland is ensured by the high annual precipitation that enhances primary production and biomass (Gischler et al., 2014; Rosenzweig, 1968). On the beach, allochthonous subsidies from the adjacent ocean provide a constant and reliable nutrient input for the beach‐dwelling taxa (Paetzold et al., 2008; Stapp & Polis, 2003). In the open forest cluster, most of the co‐occurring taxa with neither positive nor negative associations to any other taxa where predatory spiders (*Myrmarachne sp*., *Clubiona sp., Neoscona sp., Linyphia sp. Carrhotus sp.,* and one unidentified spider species “Chelicerata sp. 3”). When the abundance of their herbivorous prey taxa is sufficiently high, different spiders can overlap in their occurrences without apparent interspecific competition or competitive exclusion (Chesson, 2000).

Negative coassociations were observed within the open forest (7% of all pairwise associations), dense forest (18% of all pairwise associations), and the beach cluster (17% of all pairwise associations). For example, in the beach cluster, the presence of the predatory ghost crab *O. cordimana*, a known predator of other crustaceans like *Coenobita spp*., excluded other beach‐associated organisms from the plots, as they avoid this predatory crab (Burggren & McMahon, 1988; Pringle et al., 2019). In the dense forest cluster, the ant *Tapinoma fragile* excluded several hexapods, including predatory spider taxa, a phenomenon already demonstrated in controlled manipulation experiments for other ant species (Mestre et al., 2016).

The combination of ecological niche and co‐occurrence pattern analysis for the investigation of species distribution within tropical atoll insular ecosystems of the Indo‐Pacific region revealed multiple mechanisms and levels of organization and structuring (Fox, 1981): Within the insular habitats, the different taxa co‐occur and this co‐occurrence is likely stabilized via a high availability of resources or a fine‐scale niche differentiation not detectable in the current sampling approach, for example, differences in shell resource use of the two present hermit crab species (Chesson & Warner, 1981; Darmon et al., 2012; Steibl & Laforsch, 2020). The overall distribution of species within the insular ecosystem is, however, organized in a compartmentalized way. Comparable compartmentalization has already been established for pollination networks or food webs, where compartmentalization increases overall ecosystem resilience, as disturbances spread more slowly throughout the system and between different compartments (Bastolla et al., 2009; Olesen et al., 2007; Pimm & Lawton, 1980; Tylianakis et al., 2010). Similarly, compartmentalization of niche space might be a mechanism that enhances the resilience of the ground‐associated invertebrate community, as, for example, environmental changes in vegetation cover or seagrass deposition only affect single compartments of the insular ecosystem rather than the whole community.

As the basic mechanisms of ecological organization are in general transferable from simple, that is, insular, to more complex, that is, mainland terrestrial, ecosystems (Goldstein, 1975), we suggest that our results on the organization and architecture of niche space on atoll insular ecosystems of the Indo‐Pacific region could be transferable to a wider range of terrestrial insular or mainland ecosystems. As a compartmentalized architecture is known to increase ecosystem resilience (Olesen et al., 2007), it is conceivable that niche compartmentalization might also be a more widespread phenomenon that benefits the stability of different ecosystems than previously thought.

Our findings have further important implications for ecosystem conservation. It would be relevant in future impact assessments to first identify the clusters in niche occupation of a given ecosystem and to develop specifically tailored protective measurements to conserve each cluster with its unique assembly of species. Only in this way can a conservation action plan guarantee the overall stability and protection of all organisms in an ecosystem rather than protecting just some compartments of the faunal community.

## CONFLICT OF INTEREST

The authors declare no conflicting interests.

## AUTHOR CONTRIBUTION

**Sebastian Steibl:** Conceptualization (equal); Data curation (equal); Formal analysis (equal); Funding acquisition (equal); Investigation (equal); Methodology (equal); Software (equal); Validation (equal); Visualization (equal); Writing‐original draft (equal); Writing‐review & editing (equal). **Christian Laforsch:** Data curation (equal); Funding acquisition (equal); Project administration (equal); Resources (equal); Supervision (equal); Writing‐review & editing (equal).

## Supporting information

Table S1

## Data Availability

Raw data and statistical code can be accessed via Dryad Digital Repository (https://doi.org/10.5061/dryad.63xsj3v18).
