# Supplementary material for: Compartmentalized organization of ecological niche occupation in insular invertebrate communities
Source: Ecol Evol. 2020 Nov 24;11(1):471–80. doi: 10.1002/ece3.7067 (PMC7790627; doi:10.1002/ece3.7067)
Supplement: Supplementary file 1 — Table S1 [file ECE3-11-471-s001.docx]

**Appendix Table S1:** Overview of all identified taxa and their assignment in the clusters of the given niche space. One lepidopteran pupa (Lepidoptera sp.), found in one plot on one island and three different spider species (Chelicerata sp. 1: found in three plots on two islands; Chelicerata sp. 2: found in one plot on one island; and Chelicerata sp. 3: found in one plot on one island) could not be identified to genus/species level because they were all sub-adult or juvenile specimens and were included as Lepidoptera sp. and Chelicerata sp. 1 – 3.

| **Cluster** | **Taxon** |  |
| --- | --- | --- |
| 1 (“beach cluster”) | *Cardisoma carnifex* |  |
|  | *Coenobita perlatus* |  |
|  | *Coenobita rugosus* |  |
|  | *Grapsus sp.* |  |
|  | *Ligia dentipes* |  |
|  | *Metopograpsus messor* |  |
|  | *Natica sp.* |  |
|  | *Neritus sp.* |  |
|  | *Ocypode ceratophthalmus* |  |
|  | *Ocypode cordimana* |  |
|  | *Pachygrapsus minutus* |  |
|  | *Talitrus sp.* |  |
|  | *Trachyopella collinella* |  |
| 2 (“open forest cluster”) | *Argyrodes sp.* |  |
|  | *Camponotus compressus* |  |
|  | *Carrhotus sp.* |  |
|  | Chelicerata sp. 3 |  |
|  | *Chilochorus subindicus* |  |
|  | *Clubiona sp.* |  |
|  | *Ctenolepisma sp.* |  |
|  | *Labiduria riparia* |  |
|  | *Linyphia sp.* |  |
|  | *Myrmarachne sp.* |  |
|  | *Neoscona sp.* |  |
| 3 (“dense forest cluster”) | *Anoplolepis gracilipes* |  |
|  | *Araneus sp.* |  |
|  | *Balta sp.* |  |
|  | Chelicerata sp. 1 |  |
|  | Chelicerata sp. 2 |  |
|  | *Crassopriza lyoni* |  |
|  | *Cubaris sp.* |  |
|  | *Dysdercus cingulatus* |  |
|  | *Elasmolomus pallens* |  |
|  | *Eucarlia hofmanni* |  |
|  | *Heteropoda venatoria* |  |
|  | *Lobopterella dimidiatipes* |  |
|  | *Pycnoscelus indicus* |  |
|  | *Rhysida longipes* |  |
|  | *Sason robustum* |  |
|  | *Tapinoma fragile* |  |
|  | *Teleogryllus mitratus* |  |
| 4 (“grassland cluster”) | *Acrosternum gramineum* |  |
|  | *Diabolocatantops innotabilis* |  |
|  | *Diaphorina citri* |  |
|  | *Diplacodes trivalis* |  |
|  | *Draposa lyrivulva* |  |
|  | *Garypus maldivensis* |  |
|  | *Gonocephalum lewisi* |  |
|  | Lepidoptera sp. (pupa) |  |
|  | *Platymetopus flavilabris* |  |
|  | *Plexippus cf. paykulli* |  |
|  | *Tenebrio sp.* |  |
|  | *Thomisus pugilis* |  |
